# The Sign Problem in Density Matrix Quantum Monte Carlo

**DOI:** 10.1021/acs.jctc.1c00078

**Published:** 2021-09-21

**Authors:** Hayley R. Petras, William Z. Van Benschoten, Sai Kumar Ramadugu, James J. Shepherd

**Affiliations:** † Department of Chemistry, University of Iowa, Iowa City, Iowa 52242-1294, United States

## Abstract

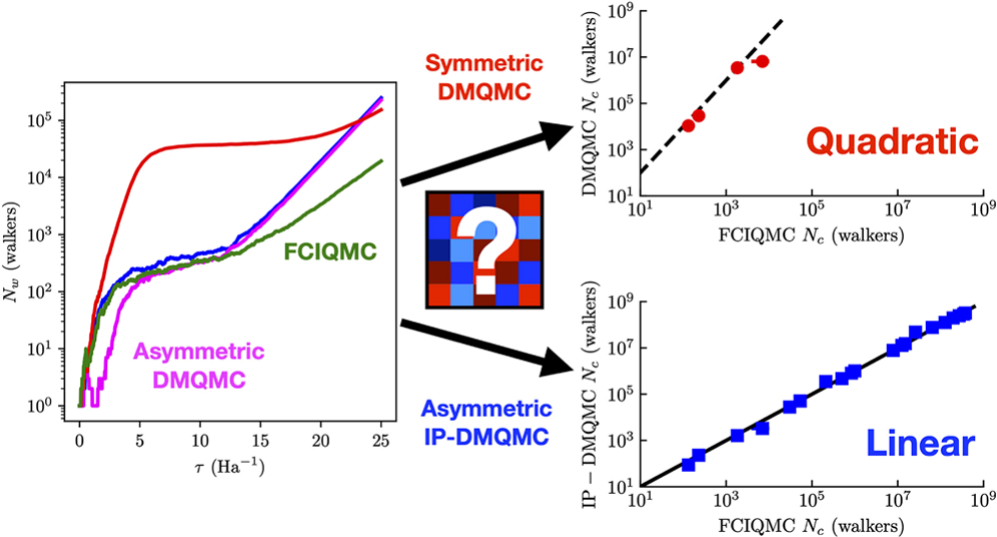

Density matrix quantum Monte Carlo (DMQMC) is a recently developed method for stochastically sampling the *N*-particle thermal density matrix to obtain exact-on-average energies for model and ab initio systems. We report a systematic numerical study of the sign problem in DMQMC based on simulations of atomic and molecular systems. In DMQMC, the density matrix is written in an outer product basis of Slater determinants. In principle, this means that DMQMC needs to sample a space that scales in the system size, *N*, as O[(exp(N))^2^]. In practice, removing the sign problem requires a total walker population that exceeds a system-dependent critical walker population (*N*_c_), imposing limitations on both storage and compute time. We establish that *N*_c_ for DMQMC is the square of *N*_c_ for FCIQMC. By contrast, the minimum *N*_c_ in the interaction picture modification of DMQMC (IP-DMQMC) is only linearly related to the *N*_c_ for FCIQMC. We find that this difference originates from the difference in propagation of IP-DMQMC versus canonical DMQMC: the former is asymmetric, whereas the latter is symmetric. When an asymmetric mode of propagation is used in DMQMC, there is a much greater stochastic error and is thus prohibitively expensive for DMQMC without the interaction picture adaptation. Finally, we find that the equivalence between IP-DMQMC and FCIQMC seems to extend to the initiator approximation, which is often required to study larger systems with large basis sets. This suggests that IP-DMQMC offers a way to ameliorate the cost of moving between a Slater determinant space and an outer product basis.

## 1 Introduction

In a recent study, we showed that the density matrix quantum Monte Carlo (DMQMC) method could be applied to molecular systems, extending it beyond its original applications to model systems in condensed matter physics.^1^ Finite-temperature electronic structure methods are becoming increasingly important in applications such as plasmonic catalysis,^2,3^ the study of planetary interiors,^4^ and solid-state materials.^5^ In these applications, modeling temperature dependence is essential to obtain physical and chemical properties, such as phase diagrams and excitation energies. The inclusion of temperature in quantum chemistry methods is difficult because, at finite temperatures, more than one state is often occupied, increasing the difficulty of solving the Schrödinger equation. DMQMC joins a growing set of methods attempting to solve the finite-temperature problem that have attracted recent attention among quantum chemists, including other quantum Monte Carlo methods,^6−11^ many-body theories,^12−14^ and others.^15−19^ Many of these methods, like DMQMC, continue to undergo development at the time of this publication.^20−28^

Widespread adoption of all methods in the FCIQMC family, including DMQMC, is hindered, in part, due to the sign problem. In FCIQMC-based methods, coefficients in the wave function (or density matrix in the case of DMQMC) are sampled by a distribution of walkers. The original FCIQMC paper found that simulations exceeding a critical walker population were able to successfully resolve the sign of the wave function and generate an exact-on-average energy estimate; it was not possible to find accurate estimates of the energy from simulations containing populations lower than this plateau.^29^ FCIQMC employs a discrete basis set, which means that walkers arriving at the same site can be exactly annihilated. A simulation with a growing walker population will have its growth briefly stall out, forming an “annihilation plateau” in the total walker population (*N*_w_) as a function of the simulation iteration as the simulation establishes the sign of critical elements of the wave function. When the population has grown above the plateau, the sign problem is resolved, and exact energies can be collected in a straightforward manner.

The sign problem in FCIQMC was discussed in depth in the early developmental papers^29,30^ for the method before subsequently being systematically studied by Spencer et al.,^31^ whose work we refer to throughout. This work showed that the sign problem is related to the growth of an unphysical dominant solution in the positively and negatively signed walker populations, causing significant noise unless walker populations are high enough to allow for sufficient annihilation. There have been attempts to fix the sign problem by leveraging this understanding directly, using a fixed-node or trial wave function approach.^28,32^ The initiator approach in FCIQMC removes the annihilation plateau at the cost of introducing a small error in the energy (which can be removed by increasing the number of walkers in the simulation).^33^ The motivation for and derivation of this approximation were ultimately related to the alleviation of the sign problem, allowing FCIQMC to be used to study a much broader scope of applications than it originally could in its canonical form.^30,34,35^ The development of the initiator approximation in DMQMC achieved a similar outcome, allowing us to apply it in our previous work on the uniform electron gas and simple ab initio molecular systems.^1,36^ Sign problem aside, there has recently been a slate of other improvements to FCIQMC (or FCIQMC-like) methods that are beyond the scope of this work to review in detail.^37−52^ Additionally, large-scale implementations of FCIQMC and related methods have also been recently developed; several recent papers that review current challenges and developments are provided for the interested reader.^53,54^

Here, we conduct a systematic investigation of the sign problem in density matrix quantum Monte Carlo (DMQMC). We find that the annihilation plateau in DMQMC comes from the same unphysical Hamiltonian as in FCIQMC. We measure these critical walker populations for a set of test systems from the FCIQMC literature and find that the DMQMC plateau heights are proportional to the square of the FCIQMC plateau heights. We then show that by moving to the interaction picture (IP-DMQMC), the DMQMC plateau heights scale linearly with the FCIQMC plateau heights. Unfortunately, using IP-DMQMC is not perfect: despite being able to control the sign problem, using IP-DMQMC can result in a collapse of the trace population, as unlike with FCIQMC, DMQMC has no global estimator for the energy. To address this collapse, we examine the initiator adaptation, showing that it has a similar performance as the initiator adaptation in ground-state FCIQMC calculations.

We find that the reason that IP-DMQMC has this plateau height reduction is that the propagation is asymmetric. Comparing asymmetric DMQMC to IP-DMQMC, we find that the critical populations are *the same* when a shift is used in DMQMC. While asymmetric DMQMC does appear to have cost savings in the required population compared to symmetric DMQMC, this is offset by the need to sample over the rows (or columns) of the density matrix or, equivalently, β-loops. We believe that this shows that IP-DMQMC is as effective at computing finite-temperature energies as FCIQMC is at computing zero-temperature energies. We see this work as complementary to our previous and future studies that develop and apply DMQMC, as well as the related work of Rubenstein et al. discussing the sign problem for finite-temperature auxiliary field quantum Monte Carlo.^6,7,55^

## 2 Methods

In this section, we provide a summary of the methods used here. We begin with the three methods primarily used in this work: DMQMC, interaction picture DMQMC (IP-DMQMC), and FCIQMC. We then describe the initiator adaptation of DMQMC. We note that hartree atomic units are used throughout this paper.

### 2.1 Density Matrix Quantum Monte Carlo

We begin with the original formulation of DMQMC.^56^ Starting with the unnormalized thermal density matrix
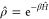
1where *Ĥ* is the Hamiltonian operator and β = (*k*_B_*T*)^−1^, we can show that the density matrix satisfies the symmetrized Bloch equation
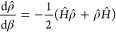
2by differentiating ρ̂(β) with respect to β. A finite difference approach with a time step Δβ can then be used to find the density matrix at any β, following
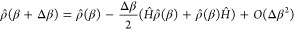
3

We can rewrite eq 3 in a basis of outer products of Slater determinants to obtain a form for the density matrix that can be solved stochastically by evolving a population of particles through the inverse temperature regime. The result is
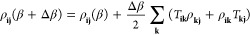
4where *T*_**ij**_ = −(*H*_**ij**_ – *S*δ_**ij**_) is the update matrix, and *S* is a variable shift for population control of the particles in the simulation, detailed later in this section.

The matrix elements ρ_**ij**_ = ⟨*D*_*i*_|ρ̂|*D*_*j*_⟩ are represented by particles in the simulation, where |*D*_*i*_⟩ are Slater determinants in the defined finite basis set. The *i* and *j* indices begin at *i* = 0 and *j* = 0, respectively. A population of *N*_w_ particles is then used to sample elements of the density matrix by propagating eq 4 with respect to β. Integer weights were used in the original FCIQMC algorithm (which DMQMC is based on); because we wish to make direct comparison with previous results, we use integer weights in this work, as well.

The simulation starts at β = 0 because in this infinite-temperature limit, all states are equally populated and thus the initial density matrix is the identity matrix. The simulation is then propagated to the desired value of β. At each step, the population is updated following rules for spawning and death of particles, summarized below. Particles of opposite signs on each matrix element are annihilated. These steps are closely analogous to FCIQMC.^29^

There are three rules for evolving particles that can be described as follows:

- Spawning: occurs from one matrix element (ρ_**ik**_) to another (ρ**_ij_**) along both the rows and columns.
- Cloning and death: occur on single matrix elements only and are designed to increase and decrease the population, respectively.
- Annihilation: particles of opposite signs on single matrix elements are removed from the simulation.

The spawning of a new particle occurs with probability ; the sign of the new particle is calculated as sign(ρ**_ij_**) = sign(ρ_**ik**_) × sign(*T*_**kj**_). Similar equations will hold for spawning from ρ_**kj**_ to ρ**_ij_**. As can be seen, the sign of the newly spawned particle depends on both the sign of the density matrix element where the particle spawned from and the sign of the element of the update matrix *T*_**kj**_ that connects the two density matrix elements. Because of this, newly spawned particles will not always be sign-coherent, a manifestation of the sign problem. To resolve the signs of the density matrix elements, a system-dependent number of particles is required. This will be explored further throughout this work.

The cloning and death of particles occurs with probability . The population increases if sign(*T*_**ii**_ + *T*_**jj**_) × sign(ρ_**ij**_) > 0 and decreases otherwise.

Annihilation occurs on single matrix elements and is used to control both the sign problem and particle growth within the simulation. Annihilation has been shown to be key in overcoming the sign problem.^29,31^

A population control must be used, so we introduce a variable shift parameter *S* that is updated according to
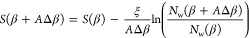
5The shift update depends on *N*_w_(β), the total number of walkers at the inverse temperature β; *A*, the number of Δβ steps between shift updates; and ξ, a shift damping parameter.

The steps outlined above are repeated until the desired inverse temperature is reached. To estimate thermodynamic quantities, one must average over many independent simulations, termed “β loops”. Then, to find the energies, the following expression is used: ⟨*Ĥ*⟩ = Tr(ρ̂*Ĥ*)/Tr(ρ̂), where the numerator and denominator of this equation are sampled separately over the course of a single trajectory. The energies are then averaged over the desired number of β loops (*N*_β_). In this work, we solely use the projected estimator and not the shift estimator because the shift estimator does not converge to the finite-temperature energy in DMQMC.^56^

### 2.2 Interaction Picture DMQMC (IP-DMQMC)

The interaction picture variant of DMQMC (IP-DMQMC) was developed to overcome two sampling issues present in the original DMQMC method: (1) the initial density matrix rarely contains the states that are important in contributing to the total energy and (2) the distribution of weights fluctuates rapidly as a function of β.^57^ Replacing the density matrix with an auxiliary matrix, *f̂*, means that the simulation can be started at a noninteracting density matrix, e^–β*Ĥ*^0^^, rather than the identity matrix, providing a good first approximation to the fully interacting density matrix for weakly correlated systems. The auxiliary matrix can be written as
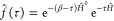
6where *Ĥ* = *Ĥ*^0^ + *V̂*, and *Ĥ*^0^ is a mean-field Hamiltonian. In this work, we use the Hartree–Fock (HF) Hamiltonian for *Ĥ*^0^, though it is possible to use a more general mean-field Hamiltonian. In practice, *Ĥ*^0^ only has diagonal matrix elements in a Slater determinant basis, and e^–(β–τ)*Ĥ*^0^^ only has diagonal matrix elements at any temperature. It is important to note that this matrix evolves from e^–β*Ĥ*^0^^ at τ = 0 to e^–β*Ĥ*^ = ρ̂(β) at τ = β, which means that IP-DMQMC only samples the correct distribution at τ = β. As such, separate simulations are required for each β value.

Differentiating *f̂* with respect to τ gives
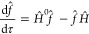
7This equation can be simulated using the rules above, with one change: the cloning/death probability in the second rule changes to *p*_d_(**ij**) = Δτ|*H*_**ii**_^0^ – *H*_**jj**_| because *Ĥ*^0^ is diagonal in the chosen basis. Whether the population increases or decreases is then based on sign(*H*_**ii**_^0^ – *H*_**jj**_) × sign(ρ_**ij**_), with the population increasing if this expression is greater than 0. In this work, IP-DMQMC uses the asymmetric spawning mode as described in Section 2.3, meaning that spawning is restricted to occur only along rows.

IP-DMQMC is the same as DMQMC, in that many simulations need to be averaged to obtain estimates for observables. When introduced, it was said that one major benefit of this variant is that as long as *H*_**ii**_^0^ > *H*_**jj**_, there is little to no death along the diagonal; this overcomes one problem with large systems in DMQMC, where the distribution along the diagonal approaches zero with β.^57^ When *H*^0^ is based on Hartree–Fock, *H*_**ii**_^0^ = *H*_**ii**_, the initial condition must also be changed; this is described in detail in the original IP-DMQMC paper.^57^ The grand canonical density matrix corresponding to *Ĥ*^0^ is used to obtain the desired distribution according to e^–β*Ĥ*^0^^.

### 2.3 Symmetric versus Asymmetric Spawning

For this study, in particular, it is important to distinguish between symmetric and asymmetric modes of spawning. In DMQMC as it is canonically formulated (eq 2), spawning is allowed on both rows and columns because the propagator is symmetric. When we refer to DMQMC in this manuscript, we generally mean this canonical formulation unless otherwise specified. However, it is also possible to have asymmetric DMQMC, with a propagator
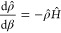
8where the spawning is restricted to rows (or equivalently, to columns). The propagator in IP-DMQMC is canonically asymmetric, with the same spawning restriction as asymmetric DMQMC. While a symmetric propagator exists for the uniform electron gas,^58^ it does not exist for molecular systems and is thus complicated to develop and test.

### 2.4 Full Configuration Interaction Quantum Monte Carlo

Next, we briefly describe the FCIQMC method,^29^ as it will be used for comparison throughout this work.

FCIQMC begins with the imaginary time Schrödinger equation
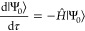
9where |Ψ_0_⟩ is the ground-state wave function, *Ĥ* is the Hamiltonian operator, and τ represents imaginary time. Here, the wave function is represented as a sum over Slater determinants |*D*_*i*_⟩
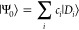
10where *c*_*i*_ is the coefficient on the *i*th determinant and the Hamiltonian is represented as
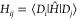
11

In the same vein as DMQMC, we can obtain a finite difference equation
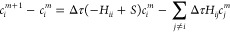
12by substituting the sum over Slater determinants (from eq 10) into eq 9, where *c*_*i*_^*m*^ is the coefficient of the *i*th determinant at iteration *m* of the simulation. Note here that the total population of particles, *N*_w_, is given by *N*_w_ = ∑_*i*_|*c*_*i*_|. To obtain an estimate of the ground-state energy, *S* is varied to keep the particle population constant and can be averaged to obtain the estimate.

At each step of an FCIQMC simulation, the particles on each wave function coefficient will undergo spawning, death/cloning, and annihilation. Particles spawn from site *i* with weight *c*_*i*_ to a connected site *j*, where *i* ≠ *j*. The spawning probability is uniform in *j*. In the death/cloning step, particles on site *i* increase or decrease their populations according to |*S* – *H*_*ii*_|Δτ. Finally, in the annihilation step, particles on site *i* with opposite signs are removed from the simulation.

The particle population is evolved using imaginary time following the rules above, through a system-dependent number of iterations. After the wave function emerges, the correlation energy is found by averaging over the iterations in the simulation.

### 2.5 Initiator Adaptation

The performance of these QMC methods can be improved through the use of the initiator approximation variation of both methods, here represented as i-DMQMC^36^ and i-FCIQMC.^33,59^ The initiator approximation works by setting a threshold *n*_add_ value, where spawning to unoccupied matrix elements only occurs from matrix elements with particle populations larger than *n*_add_, called “initiator determinants” (or from coincident spawns of particles of the same sign from two noninitiator sites). This approximation limits the number of density matrix elements (or vector elements in FCIQMC) that need to be sampled over the course of the simulation. Increasing the total number of particles, *N*_w_, can reduce the magnitude of the approximation. Both of the original algorithms are obtained as *N*_w_ → ∞. The initiator adaptation can be used with or without the interaction picture.

### 2.6 Kernel Density Estimation

The plateau height in this work is defined as the population that occurs with the highest frequency in the simulation; we call this population the critical population, *N*_c_. The Scott kernel density estimation (KDE) method^60^ was used in this work to assign critical populations through a systematic and reproducible protocol. This is a continuous adaptation from prior work.^61^ The KDE method works by calculating the probability that a certain walker population is present in the DMQMC simulation through the use of a KDE kernel, *K*. If we let *f*(*x*) be a continuous function representing the total particle population as a function of the time step in one trajectory, where *x* represents the time step in the simulation, we can use the kernel density estimator *f̂*_*h*_(*x*) to estimate the shape of the population dynamics (*f*(*x*)). The estimator is defined by
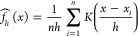
13where *h* is a smoothing parameter and *n* is the number of data points used to find the KDE kernel. The KDE kernel itself gives a probability distribution of the number of walkers as a function of the number of walkers. The maximum value of the kernel corresponds to the critical walker population.

Simulations to measure the plateau height were performed using a single β loop, and the output files were analyzed using the Python scripts provided in the HANDE software package,^53^ producing one analysis file per output file. The plateau assignments were performed on the data sets with the total walker population (*N*_w_) on a logarithmic axis. For the plateau heights in DMQMC, there were some cases where the simulations entered variable shift before the annihilation plateau occurred or the total population collapsed to zero and did not recover. If either of these situations occurred in the simulation, it was not used when measuring plateau heights.

The maximum value of the KDE kernel was assigned as the critical population *N*_c_; these are collected in a separate file. Plots of the KDE kernel and the total walker population were produced and checked visually to ensure that the critical population was assigned correctly. Once all plateaus had been validated by visual inspection, the critical populations were averaged and the standard error was calculated.

We note here that the FCIQMC critical walker populations used throughout this work are from ref (29) and were not recalculated for this work.

## 3 Results and Discussion

We performed calculations on a variety of linear hydrogen chains, and other small atoms and molecules, using the HANDE-QMC package, versions 1.4 and 1.5.^53^ All simulations in this work were performed using a time step of 0.001 and a shift damping value of 0.30. Integral dump files were generated using MOLPRO^62^ in the form of an FCIDUMP.^63^ The single-particle eigenvalues for the systems were then calculated from the orbitals in the FCIDUMP according to standard equations^64^ using an in-house code. These single-particle eigenvalues were then added to the FCIDUMP before the core Hamiltonian energy.

The equilibrium H_*n*_ chains used in this study had a bond length of 0.945110567 Å, and the stretched H_*n*_ chains had a bond length of 1.270025398 Å. These correspond to 1.786 and 2.4 au, respectively, which come from a previous study using auxiliary field quantum Monte Carlo.^6^ The H_2_O system used an O–H bond length of 0.975512 Å and a H–O–H angle of 110.565°. The CH_4_ system used a C–H bond length of 1.087728 Å and a H–C–H bond angle of 109.47122°. The bond lengths for the diatomic systems are as follows: HF, 0.91622 Å; NaH, 1.885977 Å; C_2_, 1.27273 Å; N_2_, 2.068 au, and stretched N_2_, 4.2 au. These come from a previous study using FCIQMC.^29^

The critical walker populations, or “plateau heights”, were measured using the Scott KDE method^60^ as implemented in NumPy^65^ in Python3. The DMQMC calculations to measure critical populations for the H_6_ systems were performed with initial populations of 5 × 10^2^ and target populations of 5 × 10^6^, and for H_8_, the simulations used initial populations of 5 × 10^4^ and target populations of 5 × 10^8^. These simulations were propagated to β = 25. The IP-DMQMC and FCIQMC simulations for measuring the critical populations were performed with an initial population of 1 and a target population of 5 × 10^8^. All calculations used the integer walker algorithm for all methods to maintain comparability with the plateaus reported in the first FCIQMC paper.^29^ When one walker is used, this means we are sampling exactly one row per β-loop (for asymmetric methods).

Our results are arranged as follows: in Section 3.1, we begin by confirming the presence of the annihilation plateau and compare the critical walker population in DMQMC to FCIQMC for stretched H_6_, which essentially reproduces known results from Blunt et al.^56^ In Section 3.2, we then explore the connection to the unphysical Hamiltonian related to the sign problem in FCIQMC.^31^ Next, we generalize our finding from Section 3.1 to a wide range of atomic and molecular systems in Section 3.3 and also explore the interaction picture variant of DMQMC. We then discuss similarities and differences between DMQMC and FCIQMC in Section 3.4 and energy convergence in Section 3.5. Finally, we compare the initiator adaptations to IP-DMQMC and FCIQMC in Section 3.6.

Throughout the manuscript, DMQMC refers to symmetric DMQMC. This is the only type of DMQMC discussed in Sections 3.1, 3.2, and 3.3. In Section 3.4, asymmetric DMQMC is introduced and discussed, and is used throughout the remainder of the paper. IP-DMQMC uses asymmetric propagation throughout. In section headings and the captions of figures, information about whether DMQMC is being propagated in a symmetric or an asymmetric fashion is repeated for emphasis and clarity.

### 3.1 Example of a Symmetric DMQMC Annihilation Plateau

We first begin by describing and then reproducing the original finding of the DMQMC annihilation plateau, where we offer an example of an ab initio system. This section is intended to introduce readers to salient features of an annihilation plateau. The first paper on DMQMC^56^ described the sign problem in this method as similar to that of FCIQMC due to the close similarities between the population dynamics within the methods. Of particular interest is the annihilation step, which is identical between the two methods. The annihilation step is found to be key to overcoming the sign problem, as described earlier. One difference between the two methods is that the rate of annihilation is likely less frequent in DMQMC than in FCIQMC because there are more density matrix elements than there are terms in the wave function coefficient vector. Blunt et al.^56^ suggested that because of this slower rate, a higher number of walkers would be needed in DMQMC to overcome the sign problem—approximately the square of the FCIQMC critical walker population—an observation made based on a Heisenberg model calculation.

**Figure 1:**
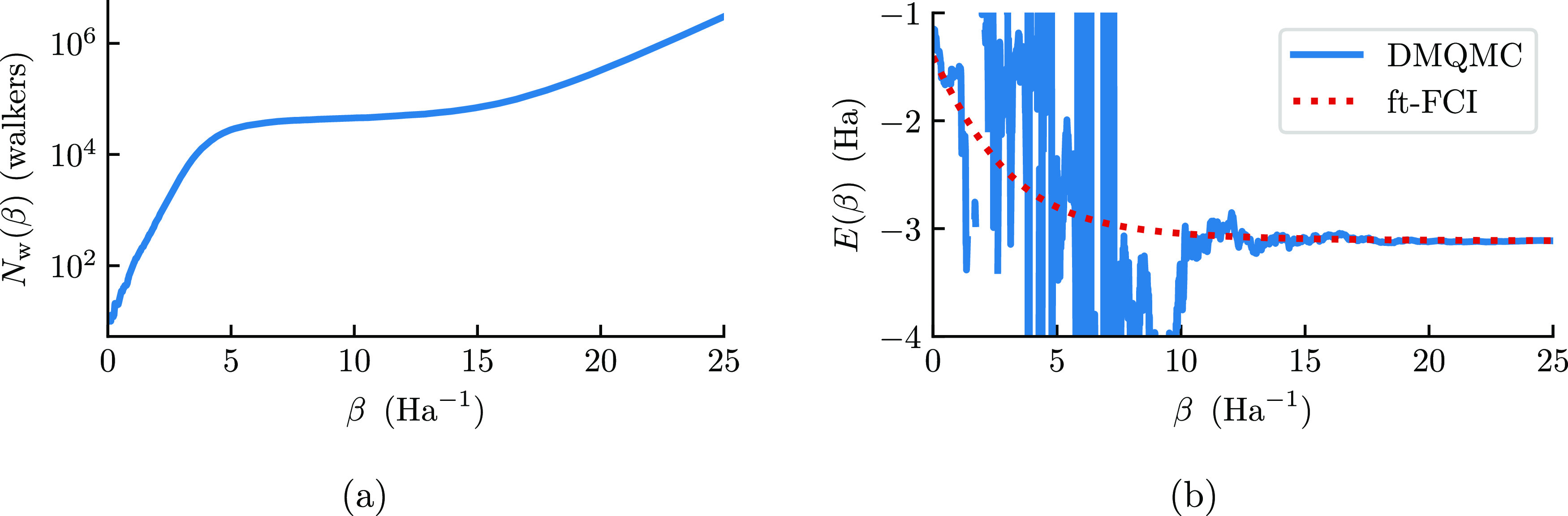
For stretched H_6_/STO-3G, (a) the total walker population, *N*_w_(β), and (b) energy, *E*(β), from a single β loop, propagated to β = 25. The simulation was started at β = 0 and used a shift of 0.343 to ensure the plateau was exited by β = 25. In panel (b), the exact diagonalization (finite-temperature full configuration interaction (ft-FCI)) is shown as a red dotted line. These results agree with prior observations.^56^ In this figure, DMQMC is symmetrically propagated.

The annihilation plateau for stretched H_6_/STO-3G is shown in Figure 1. This plateau occurs after the first exponential growth, when the population reaches a system-specific population of walkers, as seen in Figure 1a (for one β loop) between β = 0 and 5. When this specific population of particles is reached, the spawning and annihilation rates are approximately equal, resulting in no population growth—i.e., the plateau. After exiting the plateau around β = 15, we observe a second exponential growth phase. The plateau can almost always be visually identified by its distinctive appearance, although in practice, we have also automated this initial measurement of its onset (see Section 2).

When inspecting Figure 1b, the instantaneous energy estimate begins the simulation in reasonable agreement with the finite-temperature full configuration interaction (ft-FCI) energy, but quickly thereafter, the energy fluctuates considerably. After the simulation exits the plateau region, we see a return to an agreement between the DMQMC energy and the ft-FCI energy.

We can also compare the plateau heights for stretched H_6_/STO-3G (200 determinants) in DMQMC and FCIQMC. Here, the plateau height is measured as 2.927(5) × 10^4^ particles for DMQMC. This system in FCIQMC has a smaller plateau height at only 2.2(1) × 10^2^ particles. This is consistent with the description of Blunt et al.,^56^ where the authors commented that the DMQMC plateau height is approximately the square of the FCIQMC plateau height. The plateau occurs between β = 5 and 15 in this simulation, and this temperature range is not something that we can easily control as an independent variable. Thus, while the critical temperature is something we could measure, we generally neglect it for this study.

In summary, the annihilation plateau in DMQMC for ab initio systems follows previous observations based on model Hamiltonians^56^ and the FCIQMC annihilation plateau.

### 3.2 Connection between the Plateau in Symmetric DMQMC, the Unphysical Hamiltonian, and Annihilation Rate

The sign problem arises in DMQMC because spawning events are affected by the sign of the Hamiltonian, *H*_*ik*_, connecting two density matrix elements. In general, the sign of the matrix element *H*_*ik*_ (*i* ≠ *k*) can be positive or negative. One way to think about how this arises is that the application of the Slater–Condon rules brings the occupied orbitals in determinants *i* and *k* into maximum coincidence by permuting the electron indices, with each permutation causing a change in sign. It follows that ρ_*kj*_ can also have any sign. To resolve the sign of ρ_*kj*_, a sufficient number of walkers must be present to allow for the efficient cancellation of signed spawning events arriving at ρ_*kj*_.

Spencer et al.^31^ proposed that the sign problem in FCIQMC (1) was due to an unphysical Hamiltonian () whose off-diagonal matrix elements have been wholly negated while leaving the magnitude unchanged, i.e., *H̃*_*ik*_ = δ_*ik*_*H*_*ik*_ – (1 – δ_*ik*_)|*H*_*ik*_|, where δ_*ik*_ is the Kronecker delta, and (2) tended to be as severe as the energy of the dominant eigenvalue of the unphysical Hamiltonian (and the extent to which it differs from the analogous eigenvalue of the physical Hamiltonian). The authors found that these observations can be summarized by the following equation for the critical walker population *N*_c_
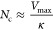
14Here, κ is the annihilation rate constant and *V*_max_ is the energy of the highest-energy eigenstate of  accounting for the shift correlation energy and the Hartree–Fock energy (i.e., *V*_max_ = *V*_0_ + *S* + *E*_HF_). This relation is only approximate because it is only valid in the limit of a small population and to first order in *V*_max_. It will also be helpful to define a variable *T*_max_ = *T*_0_ + *S* + *E*_HF_. This is the highest-energy eigenstate of *T* = −*Ĥ* shifted by the same amount as *V*_max_. Below, we test the same observations for DMQMC using the stretched H_6_/STO-3G system.

**Figure 2:**
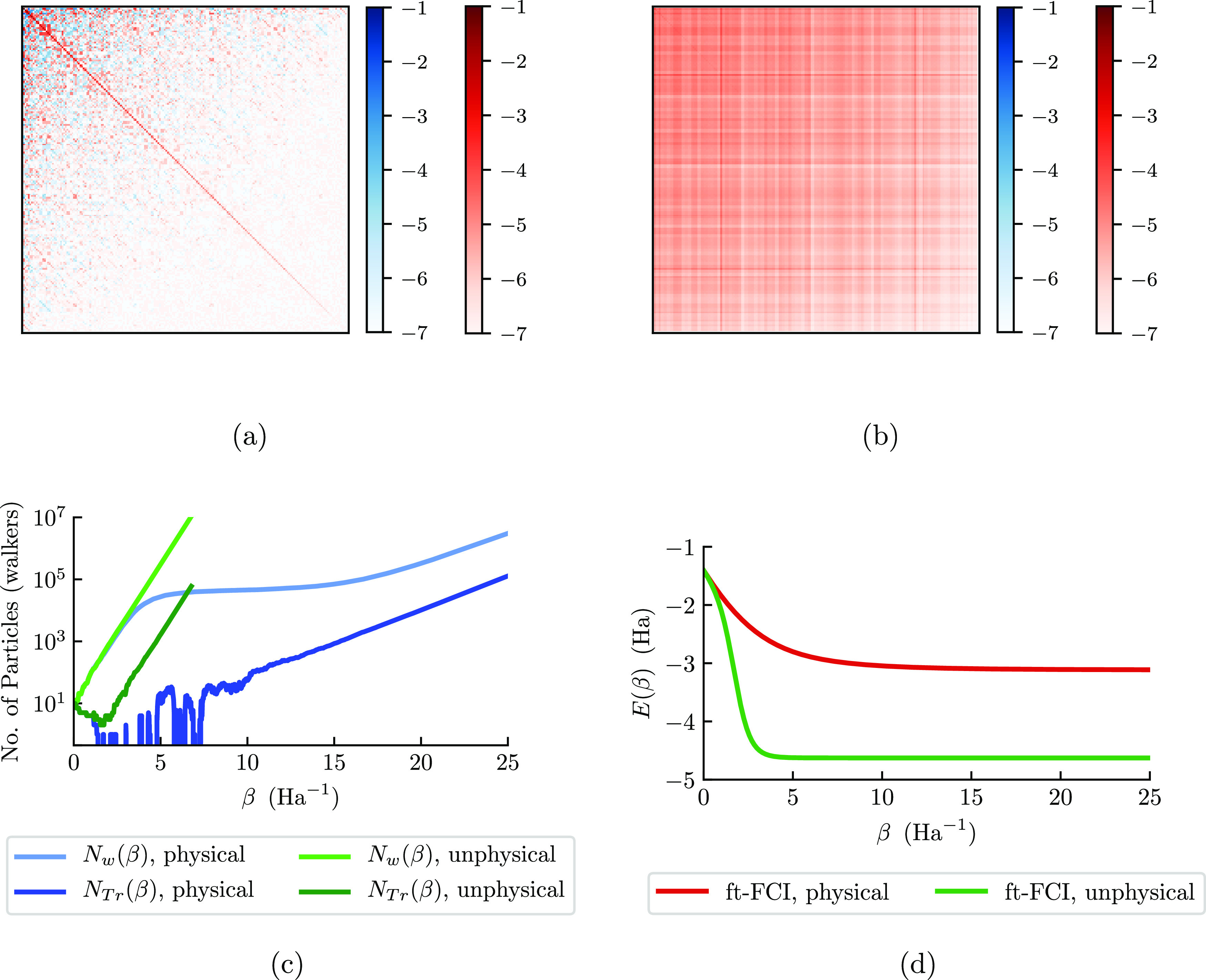
For the stretched H_6_/STO-3G system, we show the deterministic density matrices for β = 3 expressed as heatmaps for (a) the physical Hamiltonian and (b) the unphysical Hamiltonian. In panels (a) and (b), blue corresponds to negatively signed elements, and red corresponds to positively signed elements. The darker the color, the larger the weight of the element (based on a log scale, e.g., −7 represents elements of 10^–7^). In panel (c), for the same system, the population on the diagonal (*N*_Tr_) and total walker population (*N*_w_) are shown for the physical (blue) and unphysical (green) Hamiltonians from a single β loop simulation in DMQMC. In panel (d), the exact temperature-dependent diagonalizations (ft-FCI) of the physical (red) and unphysical (green) Hamiltonians are shown. In this figure, DMQMC is symmetrically propagated.

It is first useful to identify the sign structure of the density matrix for both the physical and unphysical Hamiltonians. These are shown in Figure 2a,b, respectively, for β = 3. It can be seen in this figure that these matrices differ in both the signs of their elements and the distributions of the occupied elements. In the physical Hamiltonian, there is a mixture of both positively and negatively signed elements distributed densely across the entire matrix. The combination of the heavily signed and densely packed elements explains why this inverse temperature is difficult to sample. In contrast, we see in the unphysical Hamiltonian matrix that only positively signed elements exist. Now, in the DMQMC simulations of both Hamiltonians, different dynamics are seen. For the physical Hamiltonian, DMQMC exhibits a characteristic plateau shape as the total population growth increases exponentially, pauses, and then resumes (Figure 2c). Only when the population growth resumes does the growth of walkers on the diagonal of the density matrix start in earnest. In the dynamics of the simulation, we see that the walkers on the diagonal tend to spawn and then die, depleting the diagonal population. It is only when enough of a population exists on the off-diagonal part of the density matrix and the sign structure has been established that the diagonal population can be sustained. By contrast, for the unphysical Hamiltonian, DMQMC exhibits largely uninterrupted growth in both the total population and the population of walkers on the diagonal.

To show that the sign problem is also related to the dominant eigenvector of the unphysical Hamiltonian, ft-FCI results are shown in Figure 2d. We can see here that, in general, the energies obtained from the two Hamiltonians are different, with the unphysical Hamiltonian having lower energy than the physical Hamiltonian. The one exception occurs at β = 0; at this inverse temperature, the two solutions are degenerate, as the physical and unphysical Hamiltonians have identical traces. In fact, for multiple values of β in the low β regime, the physical and unphysical Hamiltonians have similar dynamics in terms of population growth (Figure 2c), especially when compared to larger values of β. This observation is consistent with the idea that the dominant eigenvalue of the unphysical Hamiltonian causes a change in the population dynamics.

**Figure 3:**
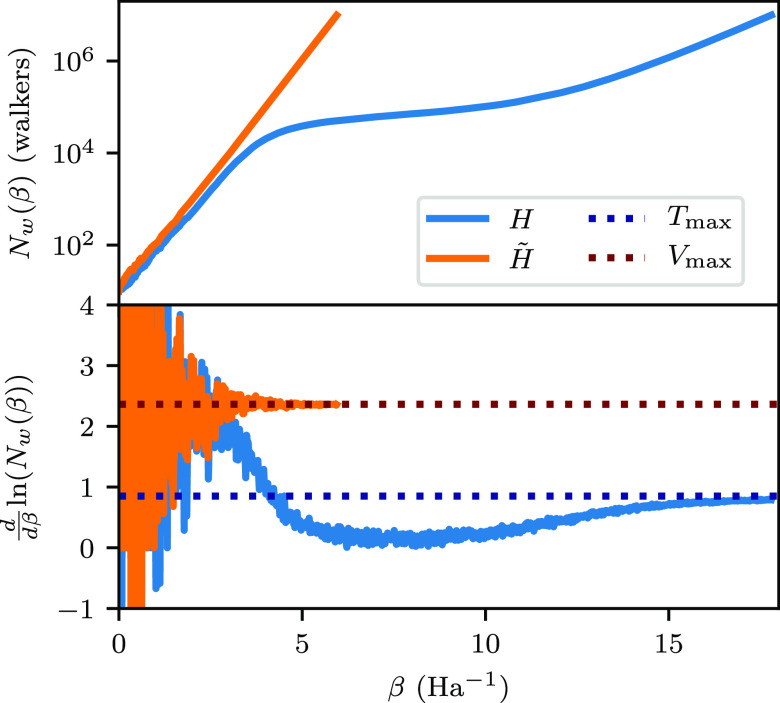
Population growth rates for DMQMC for both  and *Ĥ* for the stretched H_6_ system with a shift of *S* = 0.686. A single β loop was used. *T*_max_ and *V*_max_ were found through exact diagonalization of the respective Hamiltonian matrix. In this figure, DMQMC is symmetrically propagated.

To further analyze the role of the unphysical Hamiltonian, we can compare the population growth rates when using *Ĥ* and . Assuming a growth rate of *N*_w_ ∼ e^*k*β^, we can find the instantaneous rate constant for growth from . Plots of the number of walkers as a function of β for each Hamiltonian are shown in Figure 3. The growth rate for the  propagator oscillates around e^*V*_max_β^ for the whole of the simulation. By contrast, the growth rate for *Ĥ* in the preplateau region starts at e^*V*_max_β^, while postplateau, the growth rate tends toward e^*T*_max_β^ at large β. This lends further evidence to the relationship between the preplateau dynamics and .

**Figure 4:**
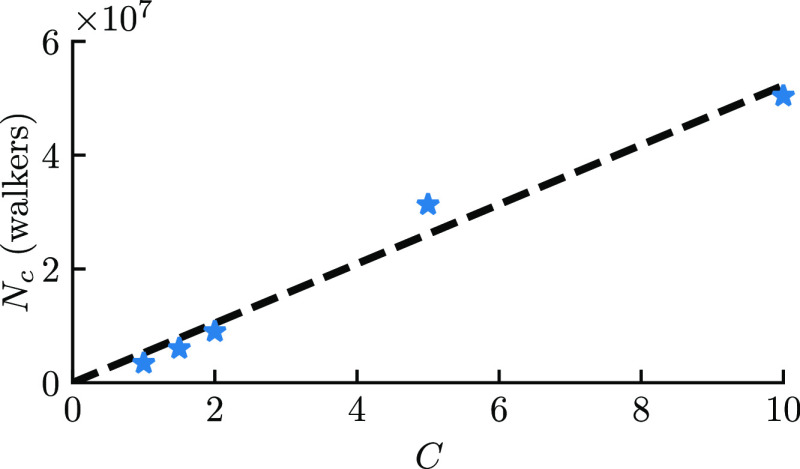
Plateau height in DMQMC (*N*_c_) for equilibrium H_8_ as a function of the scaling factor *C* described in the main text (eq 15). Here, the plateau heights shown are averages from four β loops, and the values of *C* used are 1, 1.5, 2, 5, and 10. The linear regression equation is *N*_c_ = (5.2(4) × 10^4^) × *C*, where the *y*-intercept is assumed to equal zero. In this figure, DMQMC is symmetrically propagated.

To provide further data to make the point that the dominant eigenvalue causes a change in dynamics, we scaled the off-diagonal matrix elements linearly by a positive constant *C*, starting from the true Hamiltonian, resulting in
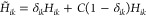
15where *H*_*ik*_ and δ_*ik*_ follow previous definitions. The plateau height follows an approximately linear trend for all *C* values tested (Figure 4), but especially for *C* ≤ 2. Thus, this fits the form of eq 14, as *V*_max_ is linear in *C* for this system at small *C* (assuming a constant κ). This observation is also consistent with that of Spencer et al.,^31^ who observed that the plateau height varies linearly with *U*/*t* in the Hubbard model, where *U* is the on-site interaction strength and *t* is the hopping integral. The analogue to the Hubbard *U* in our rescaled molecular Hamiltonian is *C*.

**Figure 5:**
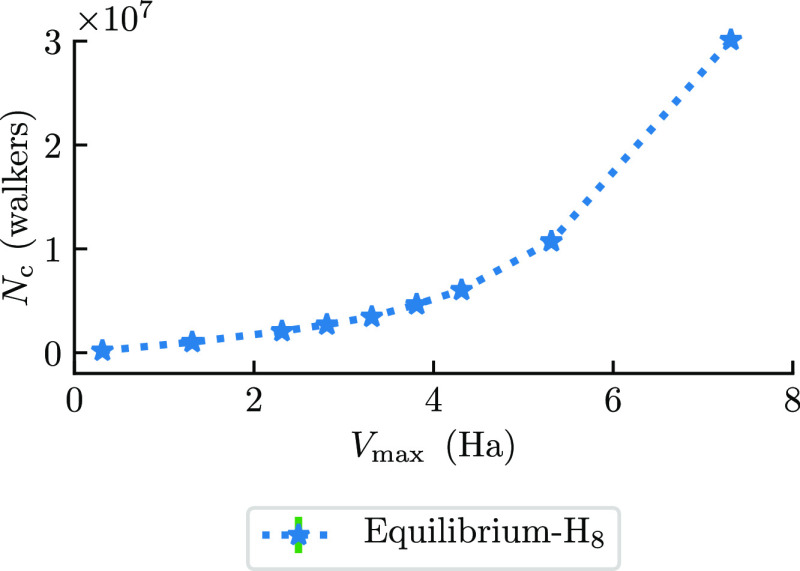
Critical population (*N*_c_, walkers) for equilibrium H_8_ as a function of the energy value *V*_max_, which includes the shift, *S*, as *V*_max_ = *V*_0_ + *S* + *E*_HF_. The critical populations here were obtained from averaging over 25 β loops. In this figure, DMQMC is symmetrically propagated.

For completeness, we also tested the final component of the plateau expression given in eq 14: the dependence on the shift parameter, *S*. We collected data for the equilibrium H_8_ system shown in Figure 5. It can be seen from these data that at low *S*, the plateau height is linear in *S*, which is consistent with the form of eq 14.

In this section, we found that the sign problem and population dynamics in DMQMC can be related to similar observations made in FCIQMC.^31^ In the next section, we explore the relationship between the plateau heights of the two methods along with IP-DMQMC.

### 3.3 How the Symmetric DMQMC and IP-DMQMC Plateau Heights Scale in Relation to the FCIQMC Plateau Height

In Section 3.1, we observed that the plateau height in DMQMC was approximately the square of the plateau height in FCIQMC for the stretched H_6_ system. In this section, we attempt to generalize this observation to a wide range of atomic and molecular systems for both DMQMC and IP-DMQMC (which was outlined in Section 2.2). As IP-DMQMC is currently limited to treat only systems with *M*_s_ = 0, we study the range of closed-shell systems that were previously considered by Booth et al.^29^ (various atoms and molecules composed of first-row atoms), supplemented with one-dimensional (1D) hydrogen chains. The latter sets are of interest because they are approximate analogues to the Hubbard models, which have been previously used for plateau height studies.^31,61^ Thus, the total test set is composed of Ne (aug-cc-pVDZ), H_2_O (cc-pCVDZ), HF (cc-pCVDZ), NaH (cc-pCVDZ), C_2_ (cc-pVDZ), CH_4_ (cc-pVDZ), N_2_ (cc-pVDZ), stretched N_2_ (cc-pVDZ), as well as stretched and equilibrium H_*n*_ (STO-3G) for even *n* between 4 and 16, inclusive. The Be atom is excluded from the test set as it has no measurable annihilation plateau in IP-DMQMC. This test set represents a variety of chemical systems, including both hetero- and homonuclear diatomics with both single and multiple bonds. Our preliminary observations indicated that the DMQMC and IP-DMQMC plateau heights were a system-dependent fraction of the size of the space, similar to FCIQMC. This made it difficult to establish a specific trend with system size.

**Figure 6:**
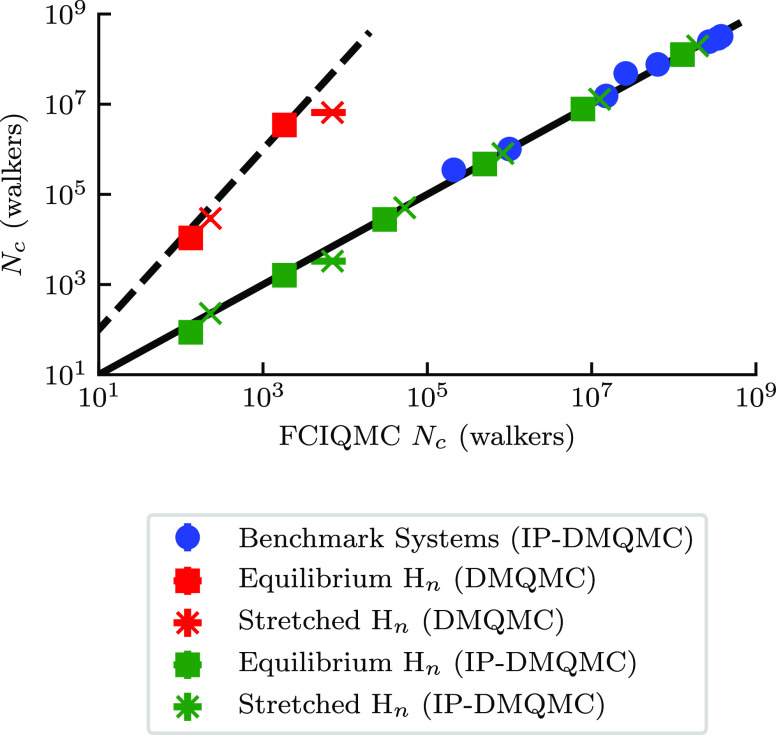
Plateau heights (*N*_c_) for DMQMC (red) and IP-DMQMC (green and blue) simulations are shown with respect to the plateau height in FCIQMC, with both axes on a logarithmic scale for the benchmark systems from Booth et al.^29^ (circle), equilibrium H_*n*_ chains (even *n* between 6 and 16 inclusive, square symbol), and stretched H_*n*_ chains (even *n* between 6 and 16 inclusive, × symbol). IP-DMQMC simulations used a target β = 25. Straight black lines are plotted for both *y* = *x* (solid) and *y* = *x*^2^ (dashed) to help guide the eye. The plateau heights were measured using the KDE method and were averaged over 25 simulations. The FCIQMC critical walker populations were obtained from published data.^29^ Error bars are shown and, in some cases, are smaller than the size of the marker. In this figure, DMQMC is symmetrically propagated, and IP-DMQMC is asymmetrically propagated.

We anticipate that each system will have a plateau height that is a system-dependent fraction of the size of the space, similar to FCIQMC. We therefore plot the DMQMC plateau height against the FCIQMC plateau height for the same system (Figure 6). These values were available for equilibrium and stretched H_6_ and for equilibrium and stretched H_8_. All of the other systems in our test set had critical populations in DMQMC that were >5 × 10^8^ particles (our choice of the cutoff in population in our experimental design). What we see in this data is that for these four systems, the DMQMC plateau height is approximately the square of the FCIQMC plateau height.

We now turn our attention to the interaction picture variant of DMQMC (IP-DMQMC). While this variant was introduced in Section 2.2, it is instructive to provide a number of methodological details at this point. IP-DMQMC targets a specific β value (here β = 25 to consistently allow the plateau to be found) and initializes on an exactly known auxiliary matrix, (*f̂*(τ) = e^–(β–τ)*Ĥ*^0^^ e^–τ*Ĥ*^), with the weights of the auxiliary matrix replacing the random sampling of the diagonal identity matrix in DMQMC. IP-DMQMC also modifies the propagator such that *f̂*(τ = β) = ρ(β) and that the propagation is asymmetric (i.e., propagation only occurs down the rows or columns of the density matrix but not both).

Figure 6 also shows plateau heights from IP-DMQMC. Our calculations in Figure 6 show that the IP-DMQMC plateau height is approximately equal to the FCIQMC plateau height for the systems studied here. For example, for the stretched H_6_ system, the IP-DMQMC plateau height is 2.2(1) × 10^2^ particles and the FCIQMC plateau height is also 2.2(1) × 10^2^ particles. This finding is remarkable, as it shows that the critical walker population in IP-DMQMC is directly related to that in FCIQMC.

**Figure 7:**
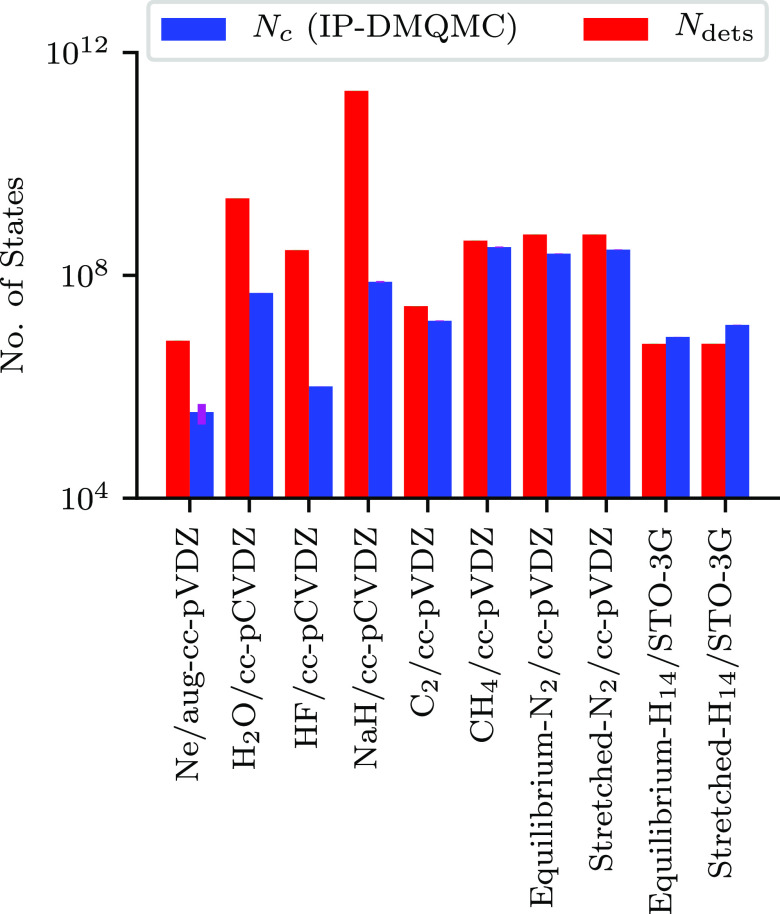
Critical population (*N*_c_, blue) in IP-DMQMC for the atoms and molecules in our test set compared to the estimated size of space (*N*_dets_, red). These *N*_c_ were collected in the same way as Figure 6. In this figure, IP-DMQMC is asymmetrically propagated.

To further emphasize this finding, Figure 7 shows the critical walker population in IP-DMQMC plotted next to the size of the Slater determinant space in FCIQMC. It can be seen that almost all of these systems have plateau heights lower than the number of determinants and, therefore, lower than the square root of the number of elements in the density matrix. How the IP-DMQMC plateau height changes with target β is explored in Appendix A.

### 3.4 How IP-DMQMC Has the Same Plateau Height as FCIQMC

**Figure 8:**
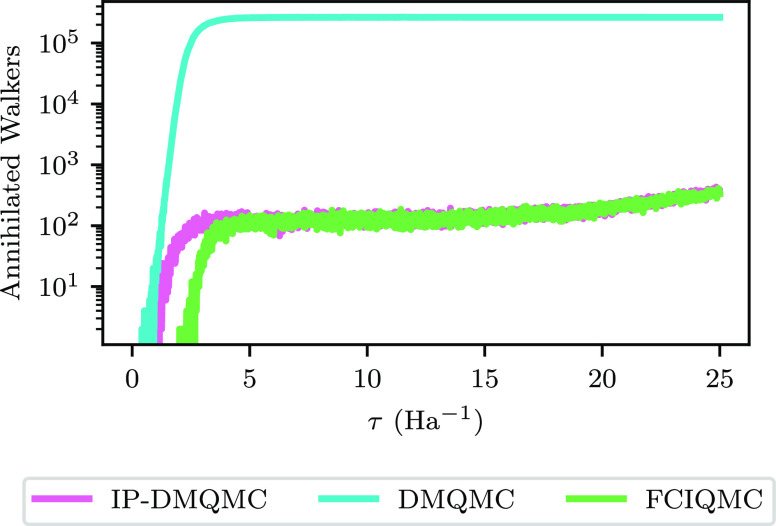
Number of annihilated walkers on a logarithmic scale in simulations of equilibrium H_8_ for FCIQMC (green), IP-DMQMC (pink), and DMQMC (blue) as a function of imaginary time (iterations for FCIQMC and β for IP-DMQMC and DMQMC). In this figure, DMQMC is symmetrically propagated, and IP-DMQMC is asymmetrically propagated.

To examine how differences in the DMQMC and IP-DMQMC methods give rise to different critical populations, we begin by analyzing eq 14. If we assume that *V*_max_ is the same (or approximately the same) in each method used, then κ can be calculated for each method. For the simulations of stretched H_6_, we can find *V*_max_ = 1.677 hartree by diagonalization. Using the plateau heights, we can then find that the κ values for FCIQMC, DMQMC, and IP-DMQMC are 7.3 × 10^–3^, 5.7 × 10^–5^, and 7.3 × 10^–3^, respectively. Here, we can see that the IP-DMQMC rate of annihilation is the same as the FCIQMC rate of annihilation and approximately the square root of that in DMQMC, i.e., IP-DMQMC requires a similar rate of annihilation as FCIQMC to resolve the sign problem. This assertion can be corroborated by measuring the growth rate of the population in Figure 2c in the large-β limit and by measuring the annihilation rate directly from the number of walkers removed in the simulation. The walkers removed by annihilation are shown in Figure 8 for the equilibrium H_8_ system. The graph shows an agreement with the observation above: the annihilation rates agree between FCIQMC and IP-DMQMC and both are much lower than the rate in DMQMC.

Going a step further, we can show additional similarities between IP-DMQMC and FCIQMC. Most notably, when IP-DMQMC is started from one walker, the propagator reduces to that of FCIQMC, exactly. To demonstrate this, we start with the IP-DMQMC propagator from Section 2
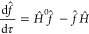
16recalling that *Ĥ*^0^ is diagonal. If we assume that our one walker lands on the zeroth row, then *f̂Ĥ*^0^ = *H*_00_*f*_00_ and will only affect the diagonal. Then, the contribution to Δ*f̂*, which is equal to *Ĥf̂*, leads to *f̂*(τ + Δτ) = (1 – Δτ*Ĥ*)*f̂*(τ), which is the FCIQMC propagator. The element *H*_00_ refers to ⟨*D*_0_|*Ĥ*|*D*_0_⟩ = *E*_HF_, the Hartree–Fock energy.

**Figure 9:**
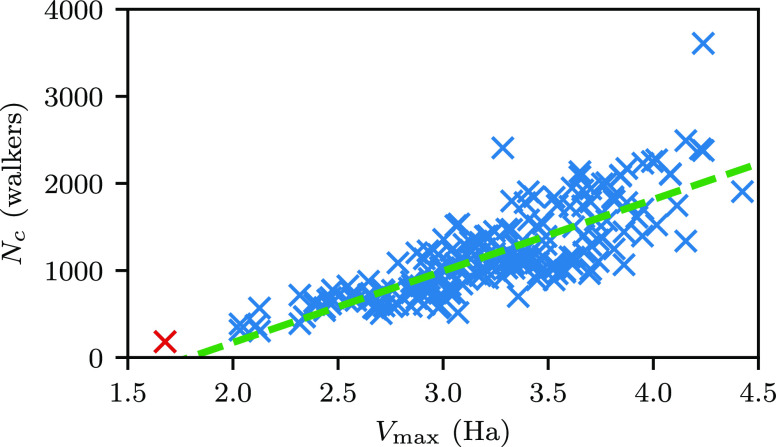
Critical population of different rows in the stretched H_6_ density matrix as a function of their *V*_max_ value. Each row has its own critical population and was measured from one β loop. The zeroth row is marked with a red × symbol. Simulations for measuring the plateau height were started with one walker and had a target population of 5 × 10^8^. *V*_max_ was calculated using an in-house analytical IP-DMQMC code by propagating *H̃* to β = 25 separately for each row. The energy was calculated at the beginning and end of the simulation, and *V*_max_ is equal to the difference between the final and initial energies. The linear fit (green dashed line) is given by *N*_w_(β) = 8.2(4) × 10^2^ (*V*_max_) – 1. 4(1) × 10^3^. In this figure, IP-DMQMC is asymmetrically propagated.

In IP-DMQMC, the term *H*_00_*f*_00_ modifies the Hamiltonian, subtracting the Hartree–Fock energy from the propagator, as in FCIQMC. This particular similarity between IP-DMQMC and FCIQMC is what guarantees the equivalence of the critical populations in Figure 6, provided that the zeroth row of *f̂* (in IP-DMQMC) is only chosen during initialization. It is reasonable to assume that when a high target β value is used (as in our simulations shown in Figure 6), the zeroth row will indeed be chosen. Thus, Figure 6 only represents a minimal plateau in IP-DMQMC when the ground-state outer product is being simulated. Unless the simulation is run at very high β, we can expect that other rows will need to be simulated. Other rows are not encountered during an IP-DMQMC simulation started from one walker because the propagator prevents other rows from being accessed during the simulation. This means that we can also measure *N*_c_ on a per-row basis. When IP-DMQMC is deliberately initialized on different rows, we find that there are slight changes in the plateau as we move away from the zeroth row. To understand these changes, we note that *N*_c_ ∝ *V*_max_ but that the effective *V*_max_ for a given row requires the −*Ĥ*^(0)^*f̂* term in the propagator is taken into account. In practice, this means that the effective *V*_max_ is increased by |*H*_*ii*_| for row *i*. The critical populations from different rows in IP-DMQMC are shown in Figure 9 for stretched H_6_, showing the linear relationship predicted by *N*_c_ ∝ *V*_max_. The average critical population (taken as an average over rows) is *N*_c_ = 1.22(3) × 10^3^, which is slightly higher than reported in the previous section (*N*_c_ = 2.2(1) × 10^2^). The larger plateau height is due to the influence of the −*Ĥ*^(0)^*f̂* term in the IP-DMQMC propagator increasing the plateau relative to *Ĥ*ρ̂, the unmodified propagator (used in DMQMC).

**Figure 10:**
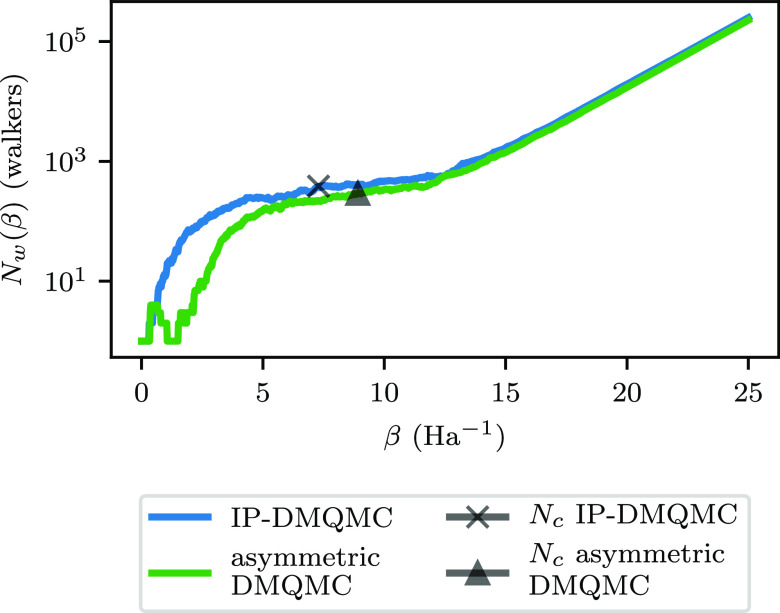
Total walker populations (*N*_w_(β)) for IP-DMQMC (blue) and asymmetric DMQMC (green) as a function of inverse temperature (β) for a random row that is not the zeroth row from Figure 9. In the asymmetric DMQMC simulation, the shift was set to *H*_*ii*_ to match the IP-DMQMC methodology. The critical populations for these IP-DMQMC and asymmetric DMQMC simulations are shown as markers for the two methods (black × symbol and black triangle, respectively). Simulations were started with one walker. In this figure, both IP-DMQMC and DMQMC are asymmetrically propagated.

One last question we consider in this section is the following: Does the interaction picture or the asymmetric propagation cause IP-DMQMC to have FCIQMC-like plateau heights? Until now, we have been running DMQMC in symmetric mode, where rows do interact because spawning occurs along rows and columns. Recall that earlier in this section, we showed that IP-DMQMC has a lower annihilation requirement than DMQMC. In practice, we find when DMQMC is run in asymmetric mode, it mirrors IP-DMQMC in having a lower plateau height than symmetric DMQMC. We actually find that the two are *identical* if the diagonal shift in asymmetric DMQMC matches what is subtracted off by *f̂Ĥ*^0^ in IP-DMQMC. This is shown by visual inspection in Figure 10. We tested the difference by running 25 β loops. We found the average plateau heights to be *N*_c_ = 3.7(2) × 10^2^ and *N*_c_ = 3.44(9) × 10^2^ for IP-DMQMC and asymmetric DMQMC, respectively, and that the difference between the two is not statistically significant. We believe, therefore, that the choice of spawning mode (symmetric versus asymmetric) gives rise to the differences we see between different methods in Figure 6.

In this section, we have determined the differences between DMQMC and IP-DMQMC that give rise to different critical populations. A key finding is that, because the critical population measurement is started from one walker, IP-DMQMC and asymmetric DMQMC only have one row occupied throughout the whole simulation (due to the structure of the propagator). For large target β, this is likely to be the ground-state-like row, making one-row IP-DMQMC equivalent to FCIQMC. For the non-ground-state rows, each can have a shift applied to make the plateau equivalent; however, without modification, the plateau grows slightly. A one-row asymmetric simulation on its own does not allow for a reliable thermal energy to be obtained. To obtain an accurate thermal energy, an average over row simulations must be found (e.g., by using β loops). The number of β loops required to converge the energy thus plays a role in the scaling of IP-DMQMC beyond the sign problem. Overall, then, this has the effect of allowing the distribution of memory costs across different β loops, perhaps allowing for the convergence of systems that are too large for symmetric propagation. However, the question remains as to how efficient this averaging is and whether there is a gain in cost relative to the DMQMC plateau. The subject of the stochastic error encountered when sampling the different rows of the density matrix is the subject of the next section.

### 3.5 Energy Convergence with Respect to Number of Rows and β Loops in IP-DMQMC and Asymmetric DMQMC

We now attempt to work out how many rows are required to converge an asymmetric DMQMC or IP-DMQMC calculation. It is, in principle, possible to converge a calculation using either row sampling (from the starting point of the simulation) or more β loops. We use an analytical implementation of IP-DMQMC and asymmetric DMQMC to measure the energy convergence of stretched H_6_ with respect to *N*_rows_ × *N*_β_ by carefully controlling the type of sampling, the number of rows, and the number of β loops. In the analytical code, walkers are initialized by randomly placing walkers (one at a time) on diagonal elements of the density matrix. A uniform random distribution is used for asymmetric DMQMC, and normalized thermal weights are used for IP-DMQMC. The propagation steps are handled deterministically, which removes the sign problem and allows us to isolate how many rows need to be sampled. The error is calculated in the normal way, using analysis tools provided in the HANDE-QMC package.

The data set for asymmetric DMQMC consisted of *N*_β_ = 2, 5, 10, 20, 50, 100, 200, 500, and 1000; *N*_rows_ = 1, 2, 5, 10, 20, 50; for β = 1–10 in integer steps. For IP-DMQMC, instead of fixing the number of rows, we fixed the number of initialization attempts at *N*_attempts_ = 1, 4, 40, 100, 300, 3000. This corresponds to approximately the same *N*_rows_ as DMQMC at β = 7. The value *N*_attempts_ can be controlled in the original HANDE implementation through walker number.

**Figure 11:**
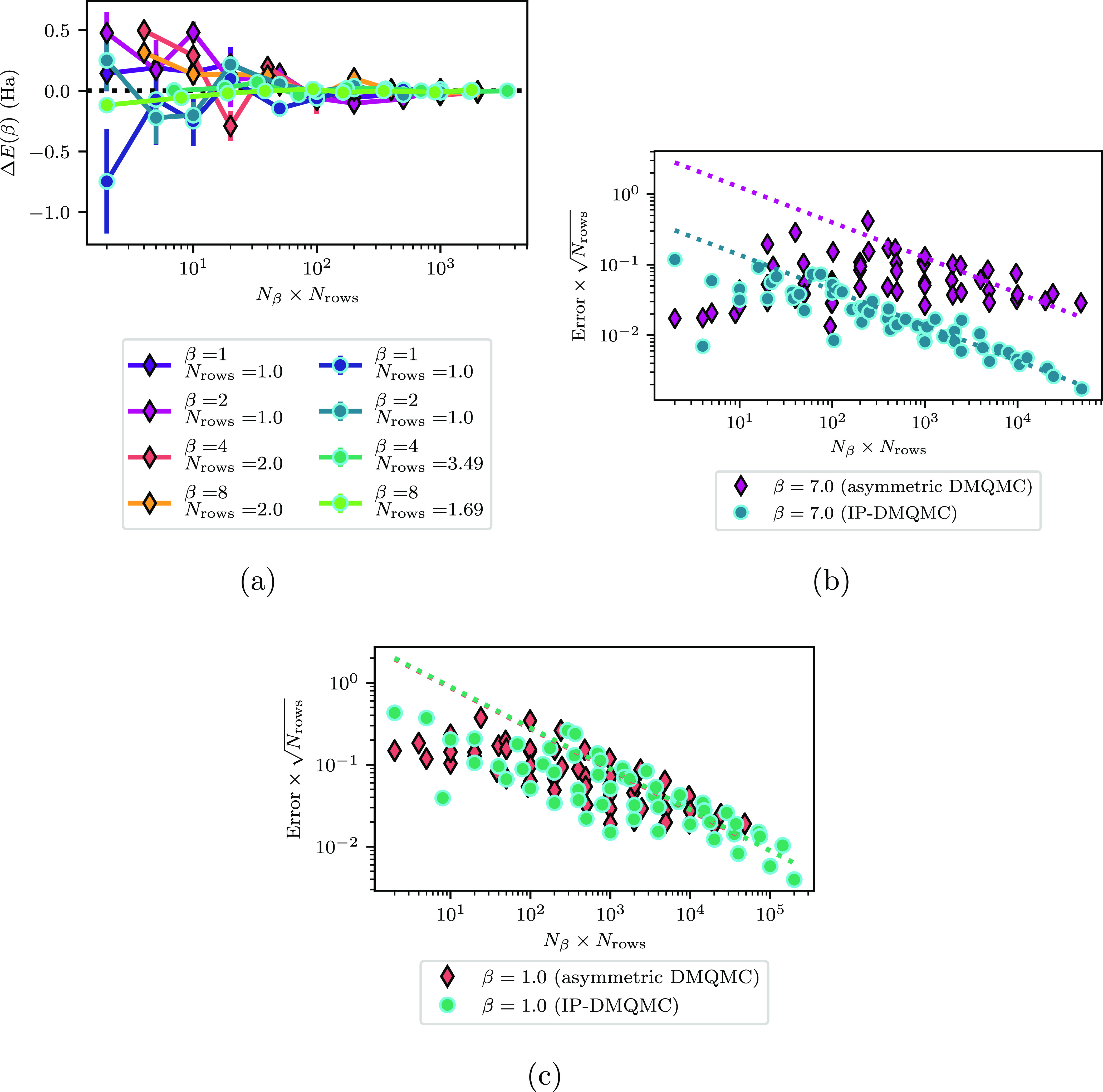
Analytical IP-DMQMC (circles with cyan outline) and asymmetric DMQMC (diamonds with black outline) simulations of stretched H_6_. (a) For a minimal number of rows, convergence to the exact energy within error is possible for all β values. (b) Stochastic error for β = 7.0 shows IP-DMQMC has a lower error than asymmetric DMQMC. (c) Stochastic error for β = 1.0 has an almost identical error between IP-DMQMC and asymmetric DMQMC. Lines of best fit depict *b* × (*N*_β_ × *N*_rows_)^1/2^. In this figure, both IP-DMQMC and DMQMC are asymmetrically propagated.

In general, the energy was well converged within error across the whole of the data set. This is, in part, because the H_6_ system contains only 200 rows (or that there are 200 FCI determinants), which means we were, in general, oversampling. However, even for *N*_rows_ × *N*_β_ < 200, we see that the energy is converged within error (<2σ) for the majority of the data set. Four exceptions with error >2σ appeared to be randomly distributed through the data set (of ∼500 points). One example of when this occurs is when a minimal number of rows is sampled, which shows that the rows can be sampled independently in IP-DMQMC and asymmetric DMQMC (Figure 11a). This is important because it at least means the memory requirement of the plateau storage (lowered due to asymmetric propagation) can be distributed across different instances of IP-DMQMC or asymmetric DMQMC, as suggested in the previous section.

In general, we found that there was a trade-off between *N*_rows_ and *N*_β_ when it came to reducing the stochastic/sampling error. This trade-off can be seen in graphs of the stochastic error plotted against *N*_rows_ × *N*_β_, where all of the data sets are (by visual inspection) part of the same distribution. This distribution generally decays according to a power-law fit of √(*N*_rows_ × *N*_β_) in the large *N*_rows_ or *N*_β_ limit. Examples of this are shown for two representative β values in Figure 11b,11c. On these graphs, the error has been multiplied by √(*N*_rows_) to make a fair comparison between IP-DMQMC and asymmetric DMQMC. In the graphs shown, we also see that β = 1.0 generally has a higher error than β = 7.0. For β = 7.0, IP-DMQMC has a lower stochastic error than asymmetric DMQMC by an order of magnitude, while at β = 1.0, their errors are more comparable. This advantage appears to be reduced at low *N*_rows_ × *N*_β_, which is the limit we want to be able to run our calculations in. It is still possible to see (in Figure 11a) that IP-DMQMC has a lower systematic error, indicating that the asymmetric DMQMC error may have an undersampling error.

Overall, we find that it is possible to take maximal advantage of the reduced plateau height of IP-DMQMC by running simulations on individual rows of the density matrix and averaging over β loops. For higher temperatures (or for asymmetric DMQMC), the whole diagonal of the density matrix must be sampled, which is likely to be costly. However, for lower temperatures (higher β), IP-DMQMC generally converges with a smaller systematic difference to the exact result and a smaller stochastic error. IP-DMQMC exhibits reduced computational cost compared to asymmetric DMQMC because it does not need to sample as many rows (whether through walkers or β loops).

### 3.6 Initiator Approach Applied to IP-DMQMC

The initiator adaptation (Section 2.5) was developed to maintain population on the diagonal. Its use is popular in FCIQMC because it removes the requirement that the simulation has to have a total walker number greater than the critical walker population (i.e., it removes the plateau) while introducing only a modest error. Unfortunately, the removal of the plateau means we cannot compare how i-FCIQMC and i-IP-DMQMC scale using this measure alone. We must, therefore, use a previous study on i-FCIQMC,^59^ where a simulation was considered converged by examining a walker population threshold measure (*N*_thresh_)—in this case, 50 000 walkers on the Hartree–Fock determinant. Measuring this threshold is analogous to measuring the plateau height because it was shown for a variety of atoms that the energy did not vary after this threshold was reached and that the simulation was converged with respect to stochastic sampling.^59^ These criteria follow the same spirit as the canonical method needing to reach a critical walker population to obtain a converged energy. We considered, but did not attempt, a “growth witness” measure introduced by other authors.^66^

**Figure 12:**
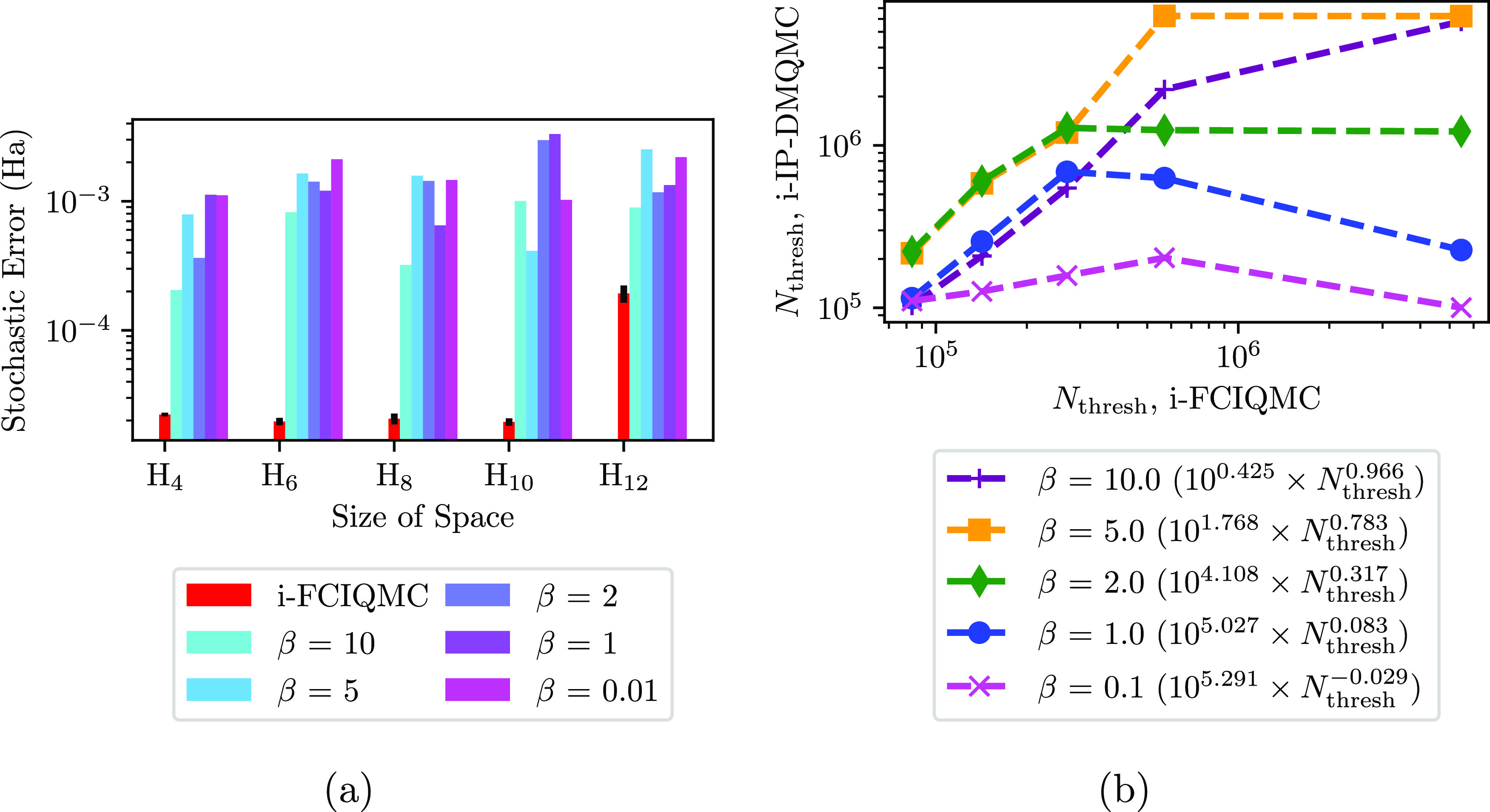
(a) Stochastic error in i-FCIQMC and i-IP-DMQMC for linear equilibrium H_*n*_ chains in the STO-3G basis set, for *n* = 4, 6, 8, 10, 12. Simulations of both methods were performed at increasing target populations starting at 100 walkers and increasing to 5 × 10^6^ walkers. For each method, a single simulation was used to determine the smallest target population (*N*_thresh_) required to reach 50 000 walkers on HF. For i-IP-DMQMC, the stochastic error shown was obtained by averaging over five β loops that reached *N*_thresh_. For i-FCIQMC, the stochastic error was found for five different simulations (50 000 report cycles, a time step of 0.001, and an initial population of 10 walkers) and then averaged. Error bars show one standard error. No shift damping was used in these simulations. (b) The total walker population at the simulation iteration at which the population threshold was met on the diagonal of the matrix (HF determinant for i-FCIQMC) in the i-IP-DMQMC simulation (*N*_thresh_) as a function of the same in the i-FCIQMC simulation (*N*_thresh_). Simulations were performed with initial populations of 10 particles and with target β values of 0.1 (magenta × symbols), 1 (blue circles), 2 (green diamonds), 5 (gold squares), and 10 (purple + symbols). The *y* = 10^*b*^ × *x*^*m*^ fits are shown in the legend. In this figure, IP-DMQMC is asymmetrically propagated.

To adapt this metric for IP-DMQMC, we consider a threshold of 50 000 walkers on the trace of the density matrix, which controls for systematic and stochastic errors simultaneously. We found that this threshold provides simulations with a mostly consistent stochastic error across system sizes (Figure 12a). In this section, we compare i-IP-DMQMC with i-FCIQMC.

In Figure 12, we show the total walker population on the diagonal of the matrix (or HF determinant for i-FCIQMC) at the simulation iteration after which the population threshold was met; we will refer to this walker value as *N*_thresh_ throughout this section. Here, we find that the i-IP-DMQMC cost in terms of walker number is the same as i-FCIQMC for low temperatures (i.e., β = 10), which makes sense because we are simulating the ground state when our choice of target β is large. In the intermediate-temperature range (β = 2 and 5), we find that for the smaller hydrogen chains, the cost is slightly higher in i-IP-DMQMC; however, as the length of the chain is increased, i-IP-DMQMC returns to being approximately the same cost as i-FCIQMC. At the two higher temperatures (β = 1 and 0.1), we find that the cost is *lower* than that of i-FCIQMC. We attribute this to the initiator adaptation itself. This variation tends to keep walkers on the diagonal of the density matrix for IP-DMQMC, and at high temperatures, more particles on the diagonal are closer to the physical solution, making convergence easier for IP-DMQMC to simulate. In general, these data show that the initiator approximation in IP-DMQMC controls the walker population in a similar manner to the initiator approximation in FCIQMC, giving us confidence that the initiator approximation can be used in future applications.

We note in passing that the importance sampling of DMQMC was also designed to maintain particles on the diagonal of the density matrix^56^ and is explored in Appendix B. We also note that we have only looked at stochastic error here; we believe that a study of systematic initiator error is extremely important going forward. Due to its complexities in the DMQMC method,^58^ a thorough examination of the systematic initiator error is left for a future study.

## 4 Conclusions

DMQMC has been shown to be a promising method for finite-temperature applications, and in this work, we have confirmed that DMQMC (especially in its interaction picture variant) shows the potential to be as effective for finite-temperature work as FCIQMC is for ground-state simulations. We confirmed that the critical walker population in symmetric DMQMC scales as the square of that in FCIQMC. Additionally, we found that the critical walker population in IP-DMQMC is the same as that in FCIQMC across all β values due to the asymmetric sampling present in IP-DMQMC. We also determined that the trade-off between sampling a small amount of rows many times versus sampling all rows fewer times is approximately equal, opening an additional avenue of development for the method. The latter is a very exciting result, as it shows that we can obtain a temperature-dependent energy at roughly the same memory and walker cost as FCIQMC, allowing us to treat systems with IP-DMQMC that cannot be treated by DMQMC. With respect to the critical walker population, this result implies that IP-DMQMC has more utility compared to DMQMC, because a smaller population of particles is required to obtain the density matrix associated with the physical Hamiltonian. Finally, we showed that the initiator adaptation with IP-DMQMC performs similarly to i-FCIQMC, again allowing us to expand upon the systems we can treat with this method.

As such, we now know that IP-DMQMC will be more useful than DMQMC for systems with a severe sign problem. One disadvantage of using IP-DMQMC, which we did not explore here, is that IP-DMQMC requires separate simulations to obtain energies for different inverse temperature values. Whether obtaining a full β spectrum in IP-DMQMC is more computationally expensive compared to that in DMQMC is still an open question. We note in passing that we did not explore the connection between this observation and the Krylov-projected FCIQMC,^67^ as we felt that this connection was beyond the scope of this work.

Overall, our results strongly suggest that the IP-DMQMC algorithm has the same potential as FCIQMC and gives a focus for future development. A natural place for future work to begin is to explore the uses (and limitations) of the initiator approach in a systematic way, as well as examining ways to modify and lower the IP-DMQMC plateau height. For example, as it is known that basis function rotations do affect the plateau height, we are inclined to explore basis functions that are optimized for a given temperature.^7^

## Figures and Tables

**Figure 13:**
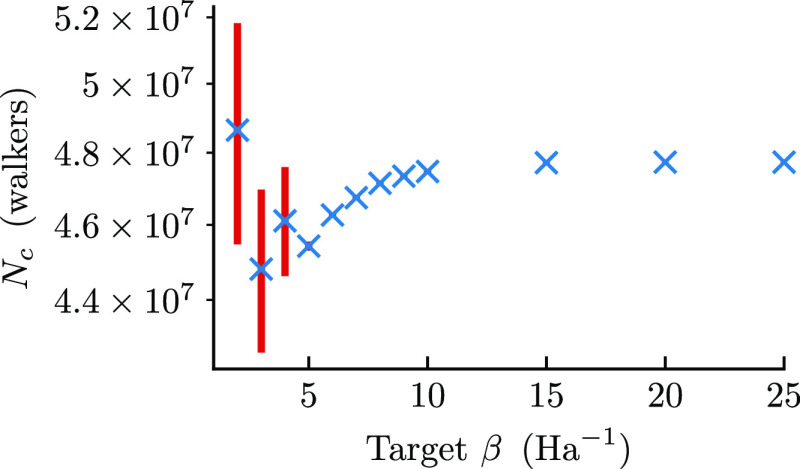
Critical population (*N*_c_) in IP-DMQMC as a function of the target β value, averaged over 10 β loops (*N*_β_) for H_2_O/cc-pCVDZ. As the β is decreased, simulations progress through the annihilation plateau to different extents, causing an increase in the stochastic error. In this figure, IP-DMQMC is asymmetrically propagated.

**Figure 14:**
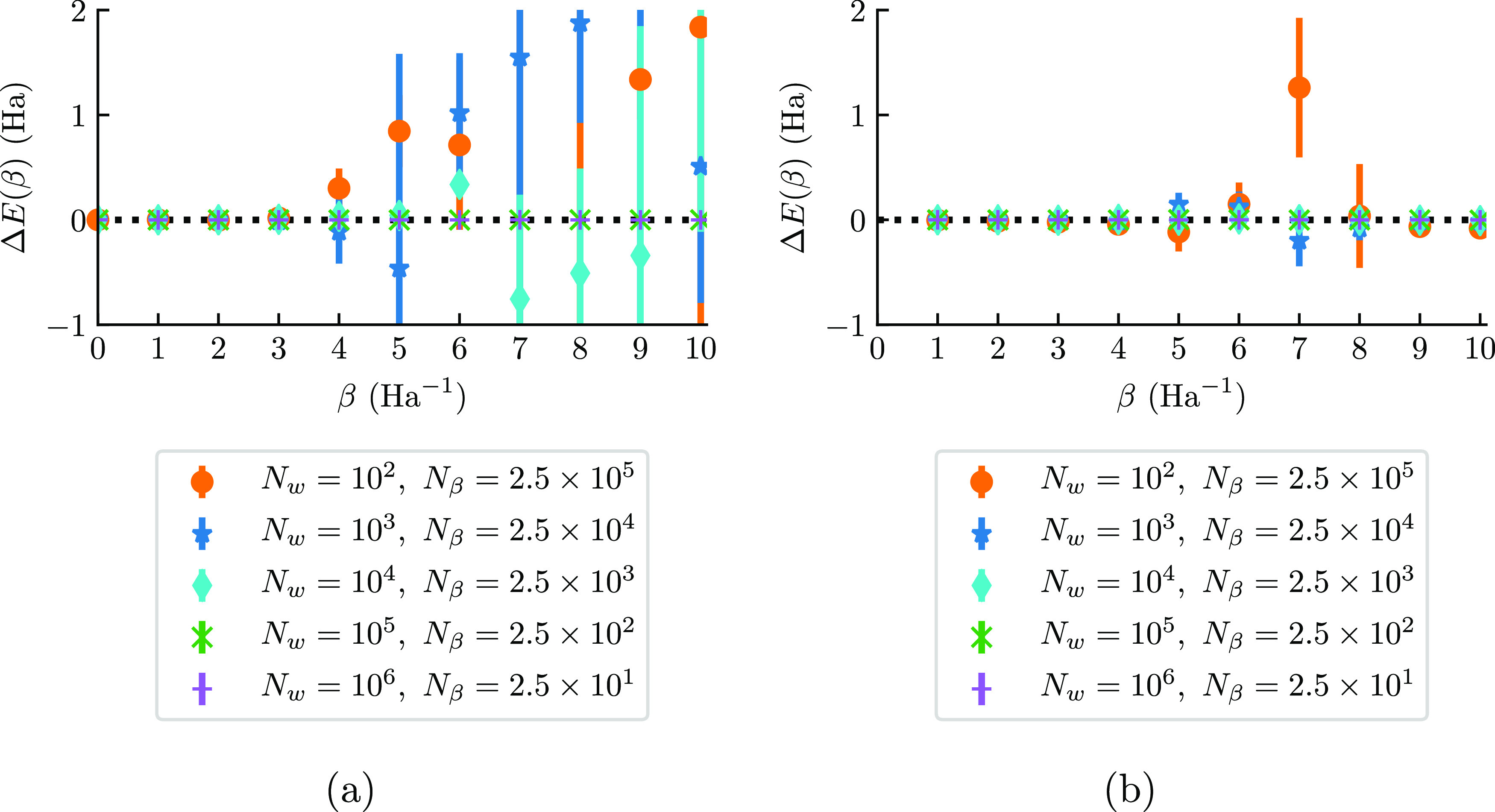
For the stretched H_6_ system, the absolute energy difference from exact diagonalization (ft-FCI) is shown for (a) DMQMC and (b) IP-DMQMC. The closer to zero the energy difference is, the more converged we consider the energy. Error bars are shown but may be smaller than the size of the marker in some cases. In both figures, five populations are shown: 10^2^ (orange circle), 10^3^ (blue star), 10^4^ (teal diamond), 10^5^ (green × symbol), and 10^6^ (purple cross). For DMQMC at *N*_w_ = 10^2^, the energy differences at β = 7 and 8 are outside the range of the plot and are −1.814 and −9.808 hartree, respectively. In this figure, DMQMC is symmetrically propagated, and IP-DMQMC is asymmetrically propagated.

**Figure 15:**
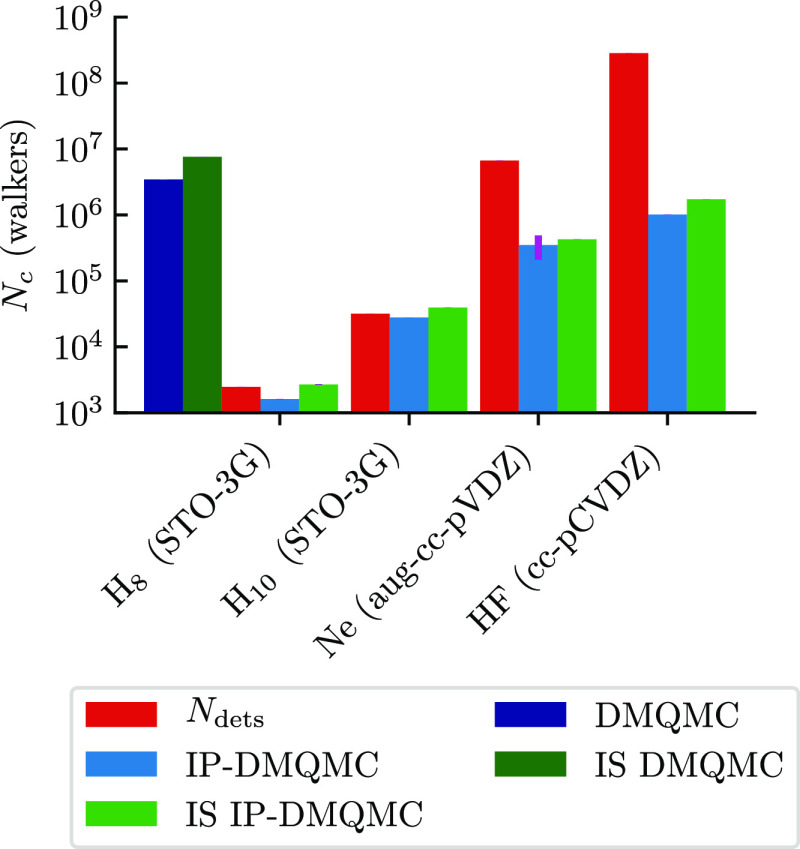
Plateau height (*N*_c_) on a logarithmic scale for equilibrium H_8_, equilibrium H_10_, Ne, and HF in IP-DMQMC (blue), IS IP-DMQMC (green), DMQMC (dark blue), and IS DMQMC (dark green). For comparison, the number of determinants (*N*_det_) for each system are shown (red). The critical populations in the importance sampling simulations were averaged over 25 β loops. Importance sampling generally increases the plateau height (with the exception of the Ne atom, where the plateau heights agree within error). In this figure, DMQMC is symmetrically propagated, and IP-DMQMC is asymmetrically propagated.

## Appendix

### Appendix A: IP-DMQMC Plateau at Different β Values

IP-DMQMC differs from DMQMC in that the former method requires unique simulations for each target β value, which must be specified at the onset of the simulation. This means that the IP-DMQMC plateau height may potentially depend on the specified β. In this section, we explore whether the IP-DMQMC plateau heights are the same as the FCIQMC plateau height across intermediate β values.

We test this potential β-dependence using the H_2_O system and present critical populations as a function of target β in Figure 13. As each target β progresses through the plateau to a varying extent by the time the target is reached, to ensure a fair test, we used a wall time limit of 4 h instead. We found that for all β values simulated, the plateau heights are between 4 × 10^7^ and 5 × 10^7^, showing evidence that the IP-DMQMC critical population is not strongly β-dependent. The FCIQMC critical population for this system was found to be 4.74 × 10^7^, and so these results confirm that the IP-DMQMC critical population is approximately the same as FCIQMC across all β values.

When moving to a different system, namely, stretched H_6_, we found that it was more challenging to measure a plateau height at intermediate β values directly, as changes in input parameters would be required to make sure that a plateau even exists by the time the target β is reached. When comparing the two systems, the walker growth is slower in the simulations of the stretched H_6_ system compared to the growth in the simulations of H_2_O. We expect that this may be why the plateau heights in H_2_O can be measured directly, whereas they cannot be measured directly in stretched H_6_. By means of an alternative, we instead study how the (symmetric) DMQMC energy converges to the exact result with walker number and how this convergence changes as a function of the target β. We do so as an alternative to changing input parameters in the IP-DMQMC simulations.

We have, so far, established that for the stretched H_6_ system the critical populations in symmetric DMQMC and IP-DMQMC (target β = 25) are 2.92(5) × 10^4^ and 2.2(1) × 10^2^ particles, respectively.

To test these values, simulations were performed at varying populations between 10^2^ and 10^6^ walkers with variable shift used throughout the simulation. We note that the random initialization algorithm of IP-DMQMC means the population can vary slightly from the starting population. For this test, the number of β loops was reduced as the walker number was increased, so the error stated is then the stochastic error for a given computational cost. By visual inspection of the energy differences to ft-FCI (Figure 14), the energy differences rapidly fall to zero above *N*_w_ = 10^5^ walkers for DMQMC and *N*_w_ = 10^3^ walkers for IP-DMQMC. After this, energies are well converged, with relatively small error bars. This is consistent with the plateaus estimated at large β referenced above. In the case of DMQMC below *N*_w_ = 10^5^ walkers, the noise grows with increasing β, which is consistent with the exponentially falling signal-to-noise ratio characteristic of the sign problem. In particular, for DMQMC simulations below the plateau, the trace becomes very small, and the energies become difficult to converge.

## Appendix B: Importance Sampling

Importance sampling was developed along with DMQMC to prevent the escape of walkers from the trace of the simulation and to improve statistical sampling; additional details can be found in ref (56). The goal of importance sampling in DMQMC is to reduce the probability of particles spawning far from the diagonal.^56^ Concentrating sampling on the diagonal matrix elements helps with the convergence of stochastic error, as the energy expression is focused on these elements. Density matrix weights stored on excitations that are more than one Hamiltonian action away from the diagonal (i.e., more distant than two-particle excitations) are less likely to contribute back to the energy directly. The approach is to give particles on higher-excitation levels larger weights to avoid changes in the expectation values of the desired operator. More particles with lower weights on or near the diagonal will help to decrease stochastic error. The number of pairs of opposite signs that must be flipped to reach |*D*_*i*_⟩ from |*D*_*j*_⟩ is defined as the excitation level, as first described by ref (56). We find that, in general, while the importance sampling approach does keep walkers on the diagonal of the density matrix, it also generally slightly increases the height of the annihilation plateau (Figure 15). We note that this finding agrees with a recent study on FCIQMC.^68^ So, while importance sampling has promise in terms of converging the energy with reduced noise (due to having an increased trace population), we do not investigate it any further here.
